# Conditional Gene Expression in *Mycobacterium abscessus*

**DOI:** 10.1371/journal.pone.0029306

**Published:** 2011-12-15

**Authors:** Mélanie Cortes, Anil Kumar Singh, Jean-Marc Reyrat, Jean-Louis Gaillard, Xavier Nassif, Jean-Louis Herrmann

**Affiliations:** 1 Université Paris Descartes, Faculté de médecine, Paris, France; 2 INSERM (U-1002), Paris, France; 3 EA 3647 Physiopathologie et diagnostic des infections microbiennes, Université Versailles St Quentin, and Laboratoire de Microbiologie, Hôpital Raymond Poincaré, AP-HP, Garches, France; Institut de Pharmacologie et de Biologie Structurale, France

## Abstract

*Mycobacterium abscessus* is an emerging human pathogen responsible for lung infections, skin and soft-tissue infections and disseminated infections in immunocompromised patients. It may exist either as a smooth (S) or rough (R) morphotype, the latter being associated with increased pathogenicity in various models. Genetic tools for homologous recombination and conditional gene expression are desperately needed to allow the study of *M. abscessus* virulence. However, descriptions of knock-out (KO) mutants in *M. abscessus* are rare, with only one KO mutant from an S strain described so far. Moreover, of the three major tools developed for homologous recombination in mycobacteria, only the one based on expression of phage recombinases is working. Several conditional gene expression tools have recently been engineered for *Mycobacterium tuberculosis* and *Mycobacterium smegmatis*, but none have been tested yet in *M. abscessus*. Based on previous experience with genetic tools allowing homologous recombination and their failure in *M. abscessus*, we evaluated the potential interest of a conditional gene expression approach using a system derived from the two repressors system, TetR/PipOFF. After several steps necessary to adapt TetR/PipOFF for *M. abscessus*, we have shown the efficiency of this system for conditional expression of an essential mycobacterial gene, *fadD32*. Inhibition of *fadD32* was demonstrated for both the S and R isotypes, with marginally better efficiency for the R isotype. Conditional gene expression using the dedicated TetR/PipOFF system vectors developed here is effective in S and R *M. abscessus*, and may constitute an interesting approach for future genetic studies in this pathogen.

## Introduction

*Mycobacterium abscessus* is an emerging, rapidly growing mycobacterium (RGM) that causes a wide spectrum of disease in humans, including chronic lung disease, skin and soft tissue disease, meningitis, meningoencephalitis, and disseminated disease in patients receiving immunosuppressive therapy [1], [2], [3]. *M. abscessus* lung disease is highly prevalent in patients with cystic fibrosis (CF) and is becoming a major issue for most CF centres worldwide [4], [5], [6]. *M. abscessus* is also a leading cause of sporadic and epidemic cases of skin and soft-tissue infections after surgery or following the use of contaminated syringes and needles [7], [8], with reports of several large outbreaks associated with injection of adrenal cortex extract, abdominoplasty, laparoscopic surgeries, mesotherapy, tattooing and piercing [9]. *M. abscessus* is one of the most antibiotic-resistant RGM species: it is naturally resistant to conventional anti-tuberculous drugs and only very few drugs are potentially active [1], [10].

Although a rapid grower, *M. abscessus* has the ability to induce a chronic disease associated with granuloma formation and on occasion caseous lesions [11], [12], [13]. Moreover, this species may exist in the form of a smooth (S) or rough (R) isotype, the latter being associated with more severe disease [4], [12], and with increased pathogenicity in various *in vitro* and *in vivo* models [13], [14], [15], [16]. With the recent availability of its complete genome sequence [17], *M. abscessus* is now a particularly relevant model for studying pathogenic mechanisms of mycobacterial disease [10]. However, research molecular microbiology and pathophysiology of diseases caused by *M. abscessus* has been hampered by the lack of genetic tools working on this agent, and the absence of knock-out mutants, with only one described so far from an S isotype [18]. Several genetic tools for mutagenesis by homologous recombination and conditional gene expression have been extensively modified and engineered for use in mycobacteria. Two thermosensitive systems [19], [20] and a plasmid encoding phage-recombinases [21] were used for homologous recombination. Several regulated expression systems were developed to control gene expression in mycobacteria. The first described expression system was based on the acetamide-inducible promoter of *M. smegmatis* [22]. Other systems used for modulating gene expression in *M. tuberculosis* were based on the TetR repressor [23], [24], [25] and revTetR [26], [27]. Alternate systems included the *Streptomyces coelicolor* pristinamycin I repressor Pip [28], the *Rhodococcus rhodochrous* nitrilase repressor NitR [29] and the recently described TetR/PipOFF double repressor system [30].

These genetics tools working on *Mycobacterium tuberculosis*, *Mycobacterium bovis* strain BCG or *M. smegmatis* are generally considered to be able to work in virtually all mycobacteria. However, this was recently found not to be the case for *M. abscessus* for the tools devoted to mutagenesis by homologous recombination. Indeed poor results were obtained with the thermosensitive counterselectable plasmid based on sucrose sensitivity [19] and the thermosensitive mycobacteriophage [20]. Only the most recently developed mycobacterial recombinase-based system [21] was used successfully in *M. abscessus* [18]. Despite this, the percentage of recombinants by homologous recombination with a double crossing-over was very low as compared to *M. smegmatis*, which still renders this tool cumbersome for the development of *M. abscessus* knock-outs [18].

Therefore research into *M. abscessus,* an emerging virulent pathogen, is still hampered by the lack of effective genetic tools. For this reason, we have considered the key role of regulated expression systems for studying mycobacteria pathogenesis, as we felt it was of outmost importance to test and adapt one of the last described repressor system in this mycobacterium. Previous experiments performed on tools for homologous recombination in our laboratory required us to modify the existing TetR/PipOFF system and to clone the different promoters in integrative vectors known to work in *M. abscessus*. We then studied the efficiency of the adapted TetR/PipOFF system firstly by controlling *lacZ* expression in *M. abscessus*, and secondly by characterizing its role as an essential gene of *fadD32* in *M*. *abscessus*. Our results have shown the difficulties encountered in adapting existing mycobacterial tools for *M. abscessus*, and we describe for the first time its success in this mycobacterium and confirmed the efficacy of the plasmid encoding phage – recombinases in creating recombinant by single cross-over in *M. abscessus*.

## Results

### Construction of TetR/PipOFF system vectors for *M. abscessus*

**Figure 1:**
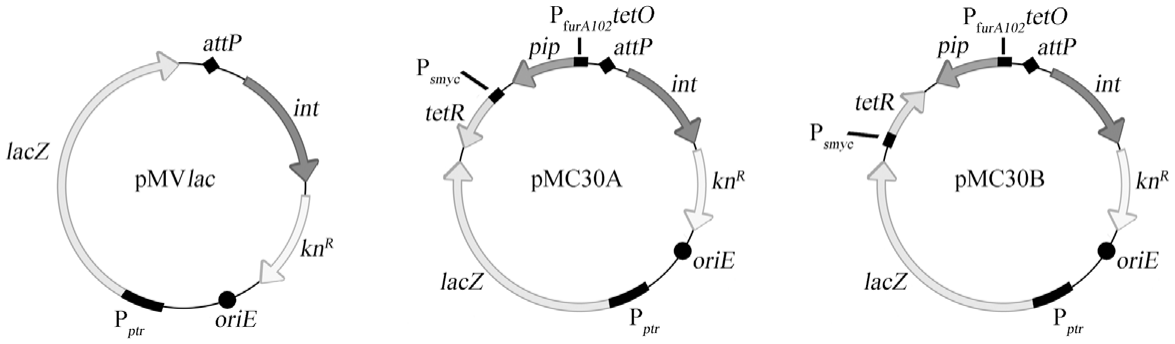
Schematic representation of plasmids pMVlac, pMC30A and pMC30B. Plasmids are derivative of the empty vector pMV306. In pMVlac, the P*_ptr_* promoter and *lacZ* was cloned in pMV306. In pMC30A or pMC30B, P*_smyc_*-*tetR* and P*_furA102_tetO*-*pip* were cloned in pMVlac in the same or opposite direction. *int*: gene coding for an integrase; *kn^R^*: kanamycin resistance gene; *oriE*: replication origin in *E.coli*; *attP*: site-specific recombination; *lacZ*: gene coding for β-galactosidase; *pip*: gene coding for the protein Pip, a repressor of the P*_ptr_* promoter; *tetR*: gene coding for TetR, a repressor of the P*_furA102_tetO* promoter in absence of ATc; P*_smyc_*: constitutive promoter; P*_furA102_tetO*: promoter repress by TetR; P*_ptr_*: promoter repress by the protein Pip.

The TetR/PipOFF repressible system was cloned into the vector pMV306, which is an integrative vector coding for the kanamycin resistance, and which has demonstrated of its efficiency in *M*. *abscessus*. Two integrative constructs were prepared and inserted in the *M. abscessus* genome: pMC30A (*lacZ*(→)*;tetR*(←)*;pip*(←)) and pMC30B (*lacZ*(→)*;tetR*(→)*;pip*(←)) (Table 1 and Figure 1). To control the potential effect the integrative plasmid had by itself we also inserted the integrative vector pMV*lac* (pMV306 with P*_ptr_*-*lacZ*) described above (Table 1 and Figure 1).

**Table 1:** Strains and plasmids used in this study.

Strains or plasmid	Relevant characteristics or sequence	Reference or source
*E. coli* DH5α		Invitrogen (Paisley, UK)
*M. smegmatis* mc^2^155		[38]
*M. abscessus* S	CIP 104536T ( = ATCC19977T), Smooth	[15]
*M. abscessus* R	CIP 104536T ( = ATCC19977T), Rough	[15]
*M. abscessus* S *fadD32* ^C^	S P*_ptr_*-*fadD32*	This study
*M. abscessus* R *fadD32* ^C^	R P*_ptr_*-*fadD32*	This study
pJV53	chec9 genes *gp60_61* under the control of the acetamidase promoter	[21]
pBSKIISK (+)	Cloning vector; Amp^R^	Stratagene
pLYG204.Zeo	Plasmid encoding a Zeo^R^ cassette	[45]
pMV306	Kn^R^, int, *attP* integrates at *attB* site on mycobacterial chromosome	[46]
pMV*lac*	pMV306 containing *lacZ* under P_ptr_ promoter	This study
pMC30A	pMV*lac* containing P*_furA102_tetO-pip* and P*_smyc_-tetR* in the same sense	This study
pMC30B	pMV*lac* containing P*_furA102_tetO-pip* and P*_smyc_-tetR* in the opposite sense	This study
pFRA42A	P*_smyc_-tetR;* P*_furA102_tetO-pip;* P*_ptr_-lacZ*; *int*; Sm^R^	[30]
pFRA61	P*_smyc_-tetR;* P*_furA102_tetO-pip* (Sm)	From R. Manganelli
pFRA50	pSM240 derivative; P*_ptr_*; (Hyg)	[30]
pMC14	pFRA50 with Zeo^R^ cassette	This study
pMC18	pMC14 with P*_ptr_*-first 953 bp of *M. abscessus fadD32*	This study

Amp^R^, ampicillin resistance; Hyg^R^, Hygromycin resistance; Kn^R^, kanamycin resistance; Zeo^R^, zeocin resistance; Sm^R^, Streptomycin resistance.

### Functionality of the TetR/PipOFF constructs in *M. smegmatis*

**Figure 2:**
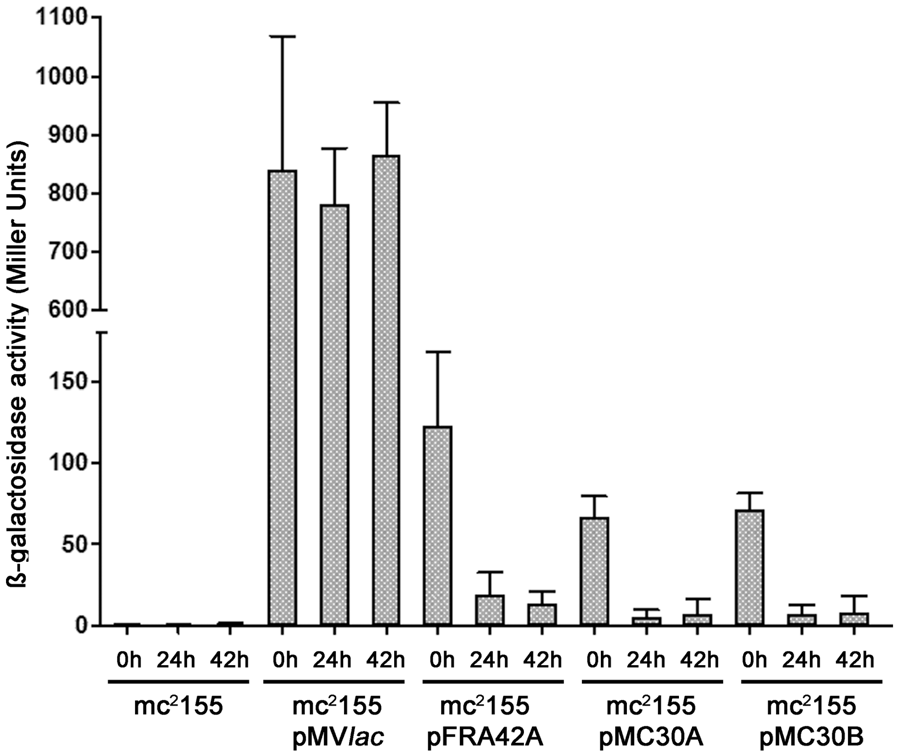
Assays with vectors pMC30A and pMC30B containing TetR/Pip OFF system in *M. smegmatis*. mc^2^155 and mc^2^155- pMV*lac,* -pFRA42A, -pMC30A and pMC30B were cultured in Luria-Bertani broth, and their respective β-Galactosidase activity was measured at 0 h, 24 h and 42 h in presence of 50 ng/ml ATc. Results were expressed in Miller units. mc^2^155 with or without pFRA42A serve as negative and positive control respectively.

pMC30A, pMC30B, pMV*lac* plasmids, and the pFRA42A (kindly provided by Boldrin et al.) [30] were electroporated into *M. smegmatis* mc^2^155 strain, to demonstrate their respective functionality (Figure S1). As a negative control, *M. smegmatis* mc^2^155 strain was shown to have no β-galactosidase activity (Figure 2). By comparison, β-galactosidase activity was obtained in each transformant, with activity ranging from 123, 67 and 71 Miller units without ATc respectively for mc^2^155-pFRA42A and mc^2^155-pMC30A or -pMC30B respectively (Figure 2). By comparison, β-galactosidase activity was high in mc^2^155-pMV*lac*, with 839±228 Miller units in absence of ATc (Figure 2). Similarly, β-galactosidase activity decreased by 12 and 10-fold respectively in mc^2^155-pMC30A and mc^2^155-pMC30B after 24 h and 42 h of incubation in presence of ATc (Figure 2). By comparison, the presence of ATc had no impact on the β-galactosidase activity of mc^2^155-pMV*lac*, with 781±95 Miller units and 864±89 Miller units after 24 h and 42 h of incubation (Figure 2).

### Functionality of the TetR/PipOFF constructs in *M. abscessus* S isotype

**Figure 3:**
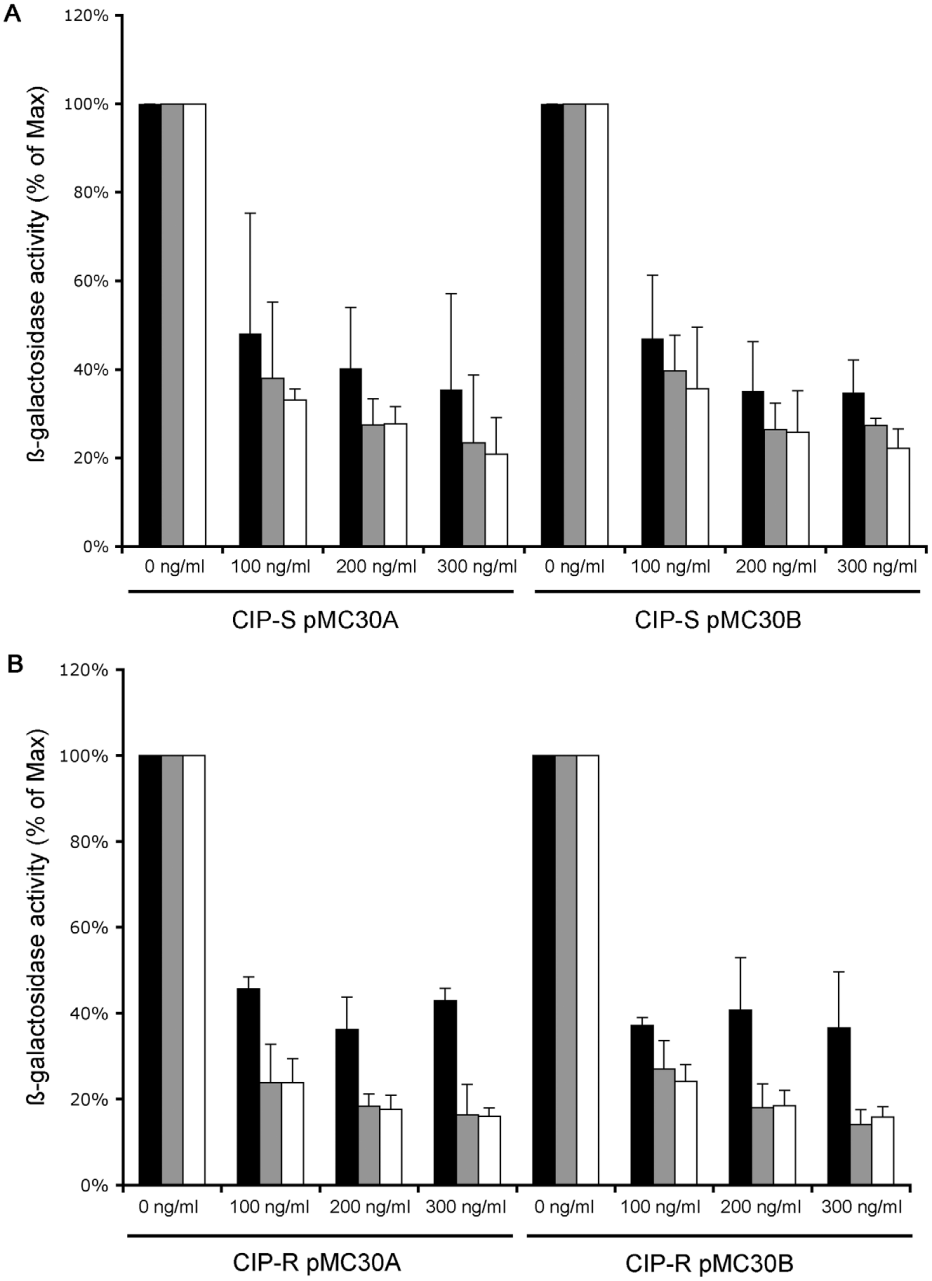
Characterization of the TetR/Pip OFF system in *M. abscessus*. *M. abscessus* S (A) and R (B) strains with pMC30A or pMC30B were grown in Luria-Bertani broth and β-Galactosidase activity was measured in absence or in presence of 100, 200 or 300 ng/ml ATc at three time points (24, 48 and 72 h). β-Galactosidase activity was expressed as the percentage of the residual activity in presence of ATc to the normal activity observed in absence of ATc. Black bars 24 h; grey bars: 48 h; white bars: 72 h.

Once the functionality of pMC30A and pMC30B were confirmed in *M. smegmatis* as compared to pFRA42A, pMC30A and pMC30B plasmids were then electroporated in *M. abscessus* strain CIP 104536T (S isotype), in addition to pMV*lac* as a positive control. β-galactosidase activity was expressed as the ratio of the activity observed in presence of ATc (100, 200 and 300 ng/ml) to the activity obtained in the absence of ATc. Such ATc concentrations were well below tetracycline MIC described towards *M. abscessus* (range 8 to 128 µg/ml) [31]. This activity was measured and expressed as a percentage at 24, 48 and 72 h. *M. abscessus* S strain had no β-galactosidase activity. β-galactosidase activity in *M. abscessus* - pMC30A S isotype was decreased by 52% at 24 h, 62% at 48 h and nearly 67% at 72 h in the presence of 100 ng/ml of ATc as compared to the β-galactosidase activity measured in absence of ATc. Increasing ATc concentrations had only a slight negative effect on the β-galactosidase activity in *M. abscessus* S isotype. β-galactosidase activity in *M. abscessus* - pMC30A S isotype was decreased by 60% fold at 24 h, 72% fold at 48 h and at 72 h at a concentration of 200 ng/ml of ATc as compared to the β-galactosidase activity in the absence of ATc. Similar results were obtained at 300 ng/ml of ATc compared to 200 ng/ml. *M. abscessus* - pMV*lac* S isotype demonstrate of a β-galactosidase activity 8 times higher compared to *M. abscessus* - pMC30A S isotype in the absence of ATc (data not shown), and kept this activity throughout the experiment (data not shown). Similar results were obtained with both integrative plasmids pMC30A and pMC30B (Figure 3A).

### Functionality of the TetR/PipOFF constructs in *M. abscessus* R isotype

The same series of experiments were performed using a well-documented R isotype from *M. abscessus* CIP 104536T [15]. β-galactosidase activity in the presence of 100 ng/ml of ATc in *M. abscessus* - pMC30A R isotype was decreased by 54% at 24 h, and 76% at 48 h and at 72 h which closely corresponded to β-galactosidase values obtained for the isotype S in the presence of 200 ng/ml of ATc (Figure 3B). In fact, the inhibition of the β-galactosidase activity in *M. abscessus* R isotype was slightly more efficient at low ATc concentrations as compared to the S isotype (Figure 3B). Increasing the ATc concentrations also increased the inhibition of β-galactosidase activity as shown by decreased values observed in the presence of 200 ng/ml or 300 ng/ml of ATc (Figure 3B). β-galactosidase activity in *M. abscessus* - pMC30A R isotype was decreased by nearly 64% at 24 h and 82% at 48 h and at 72 h respectively as compared to the β-galactosidase activity in the absence of ATc. Similar results were obtained with both integrative plasmids pMC30A and pMC30B (Figures 3A and 3B). Future described experiments will be performed using plasmid pMC30A and an ATc concentration of 200 ng/ml, where we have obtained satisfactory gene repression.

### Construction of *fadD32* conditional mutants (*fad32^c^*) in *M. abscessus* S and R isotypes

The results presented above demonstrated that the regulated TetR/PipOFF system works in *M. abscessus* S and R isotypes, with a marginally better efficiency in the R isotype. These results prompted us to test the ability of the TetR/PipOFF system to unravel essential genes by creating conditional mutants. *fadD32*, a gene involved in fatty acid biosynthesis, was chosen as a test-gene, as it has been reported to be essential in mycobacteria [28], [32]. The *fadD32* gene of *M. tuberculosis* (Rv3801c) has orthologs in *M. smegmatis* (MSMEG_6393, Blastp: 76% identities and 87 % positives) and *M. abscessus* (MAB_0179, BlastP: 66% identities and 81 % positives). Genomic organization in the *fad32* region was similar in all three mycobacterial species.

**Figure 4:**
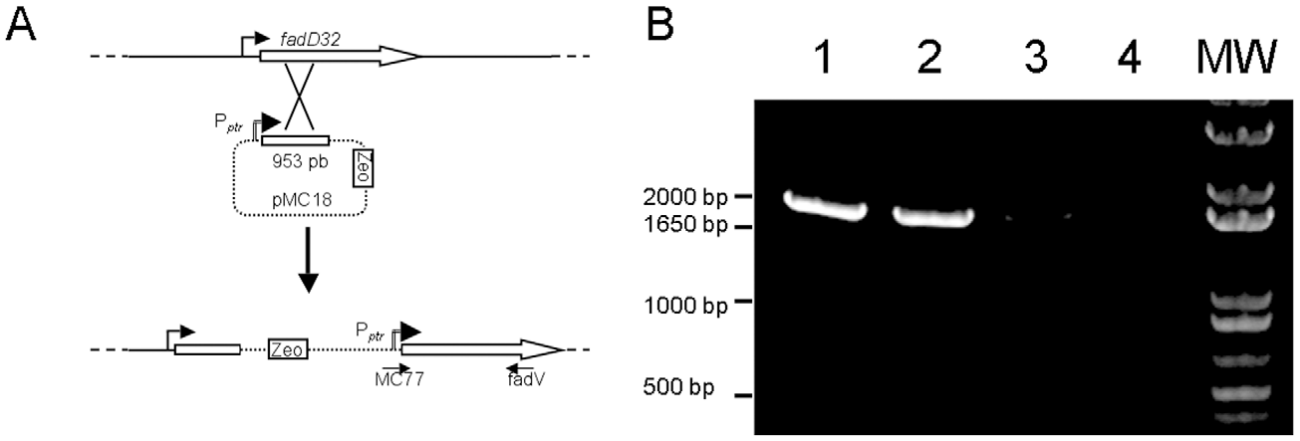
Construction of conditional mutants. (A) Schematic representation of the recombination event used to get the conditional mutant *fadD32* ^C^ after a single crossover. The size of PCR product using primers MC77 and fadV is 1700 bp. (B) PCR analysis of construction obtaining for conditional mutant. Lane 1, 2, 3 and 4: PCR with primer MC77 and fadV. Lane 1: CIP-S *fadD32* ^C^; lane 2: CIP-R *fadD32* ^C^; lane 3: CIP-S; lane 4: pMC18; lane MW: molecular size standard.

To create the conditional *fadD32* mutant in *M. abscessus*, we cloned the 5′ region of *fadD32* under the control of P*_ptr_* giving plasmid pMC18 (Figure 4A). After electroporation and zeocin selection, we checked for recombinants with a single cross-over in all the clones growing in the presence of zeocin. Such single cross-over allows replacement of the wild-type gene under the control of its own promoter by the 5′ truncated part of *fadD32* under the control of P*_ptr_* (Figure 4A). Conditional mutants were searched for by PCR using primers MC77 and fadV (Table 2) giving the expected fragment of 1700 bp. This PCR was negative in the wild type strain and on plasmid pMC18 (see Figure 4B lanes 1–4). Replacements were performed in both S and R isotypes giving strains S *fadD32* ^C^ and R *fadD32* ^C^.

**Table 2:** Primers used in this study.

Primer Name	Sequence
fadF	cgatgcatgcgttcgacaacccgttc
fadR	cgatgcatattgatgatcgagtggatgt
fadV	cggtgtattcgatgtcttg
lacZ-3	ttattattatttttgacaccagac
MC77	cgcatatgagatctccatcctgacgg
ZeoF	ccgctagctcgagcac
ZeoR	cgactagtgatccccgggaattc
MC82	agcgtgagctgctacaggac
MC83	tggatttccagcaccttctc
MC84	acatcgaatacaccgcacaa
MC85	ggattgtcgaaaaccacctg

### *fadD32* ^C^ growth characteristics in presence of ATc

**Figure 5:**
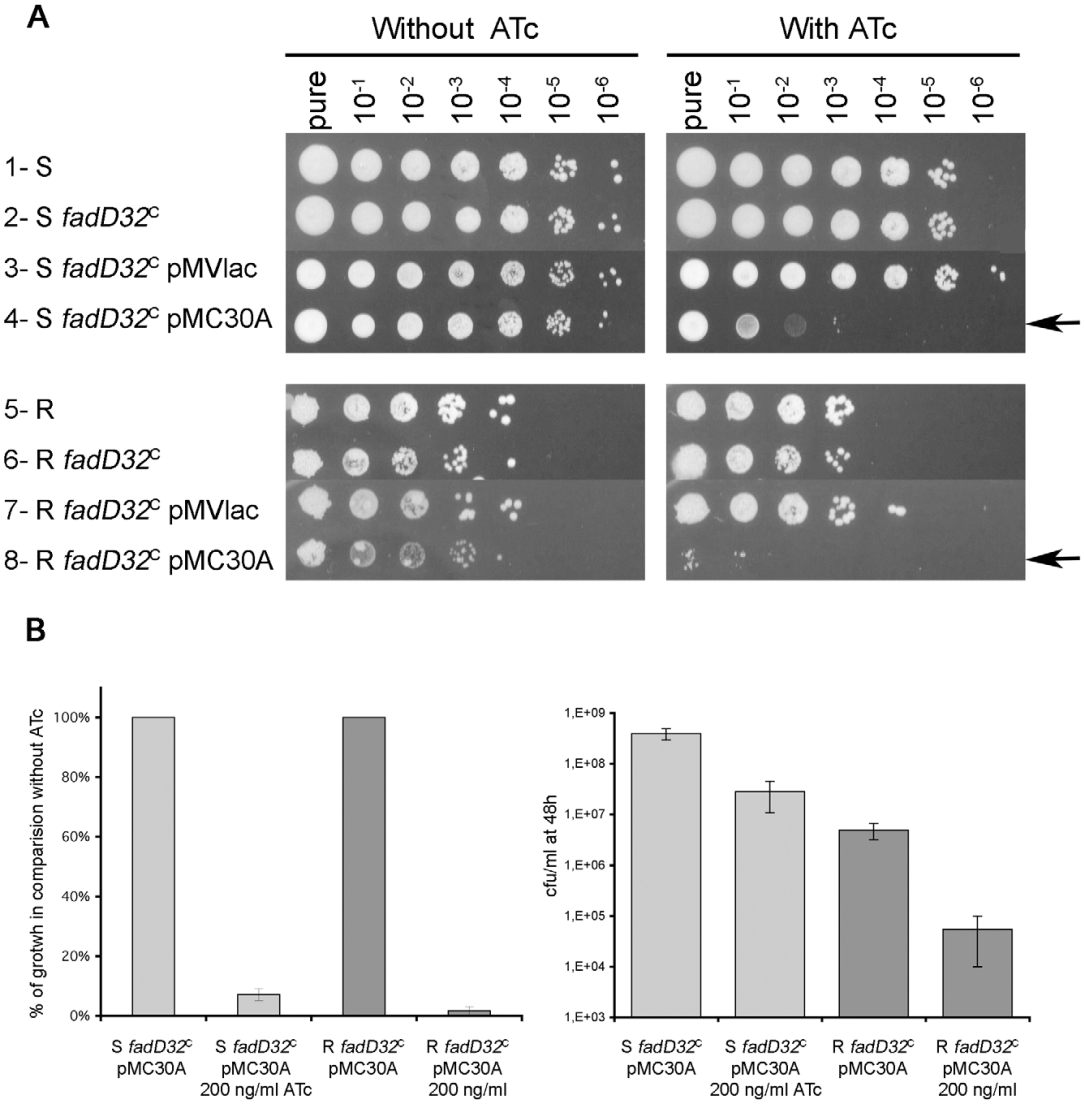
Characterization of growth for conditional mutants in *M. abscessus*. (A) Bacteria were grown to exponential phase and 5 µl of 10-fold serial dilutions were spotted onto LB agar medium supplemented or not with ATc 200 ng/ml. Plates were incubated at 37°C. Pictures were taken after five days. Arrows indicate the difference of growth in presence or absence of ATc. (B) Diluted liquid culture of S *fadD32* ^C^-pMC30A and R *fadD32* ^C^-pMC30A were separated in two cultures (one without ATc and one with 200 ng/ml ATc). After 48 h, cfu was numbered on LB agar plates. Results are expressed in percentage of growth in comparison with the same strain culture without ATc.

After transformation of plasmid pMC30A or pMV*lac* in *fadD32^C^* S and R strains, growth analysis was performed in the presence or absence of 200 ng/ml of ATc by spotting 5 µl of the liquid cultures of each conditional mutant and wild type strain (Figure 5A). The growth of wild type *M. abscessus* strain (S and R isotype) was similar with or without ATc (Figure 5A, lane 1 and 5).

For S *fadD32* ^C^ and R *fadD32* ^C^ strains containing the plasmid pMV*lac* or not, their growth was unchanged in the presence of 200 ng/ml ATc, demonstrating the correct control of *fadD32* by the P*_ptr_* promoter (Figure 5A, lane 2, 3, 6 and 7).

Strains S *fadD32* ^C^-pMC30A and R *fadD32* ^C^-pMC30A growth were similar as the wild type strain growth in absence of ATc. Reduction of growth was observed in presence of 200 ng/ml ATc for the S *fadD32* ^C^-pMC30A as shown by the absence of colonies after spotting 5 µl at dilutions 10^−2^ and 10^−3^ respectively (Figure 5A, lane 4). The absence of growth was observed for the R *fadD32* ^C^-pMC30A, as shown by the non-existence of any colonies from pure and 10^−1^ dilution (figure 5A, lane 8). Such results confirmed the inhibitory role of Pip towards P*_ptr_* promoter in presence of ATc. These experiments demonstrate the inability of *M. abscessus fad32* ^C^ to grow in the presence of ATc, demonstrating its essential role in *M. abscessus*.

Growth with or without ATc was also controlled in liquid culture. Diluted liquid culture aliquots of S *fadD32* ^C^-pMC30A and R *fadD32* ^C^-pMC30A were separated into two cultures: one without ATc and one with 200 ng/ml ATc. After 48 h, OD_600nm_ was measured and CFU counts were numbered on LB agar plates (Figure 5B). CFU counts showed that S *fadD32* ^C^-pMC30A and R *fadD32* ^C^-pMC30A growths were decreased, in the presence of ATc, by 93 and 99% of the values of the initial inocula respectively (Figure 5B). Optical density values at 600 nm were similar, with or without ATc, for both S/R *fadD32* ^C^-pMC30A variants (data not shown). These results were in agreement with data obtained on solid medium.

**Figure 6:**
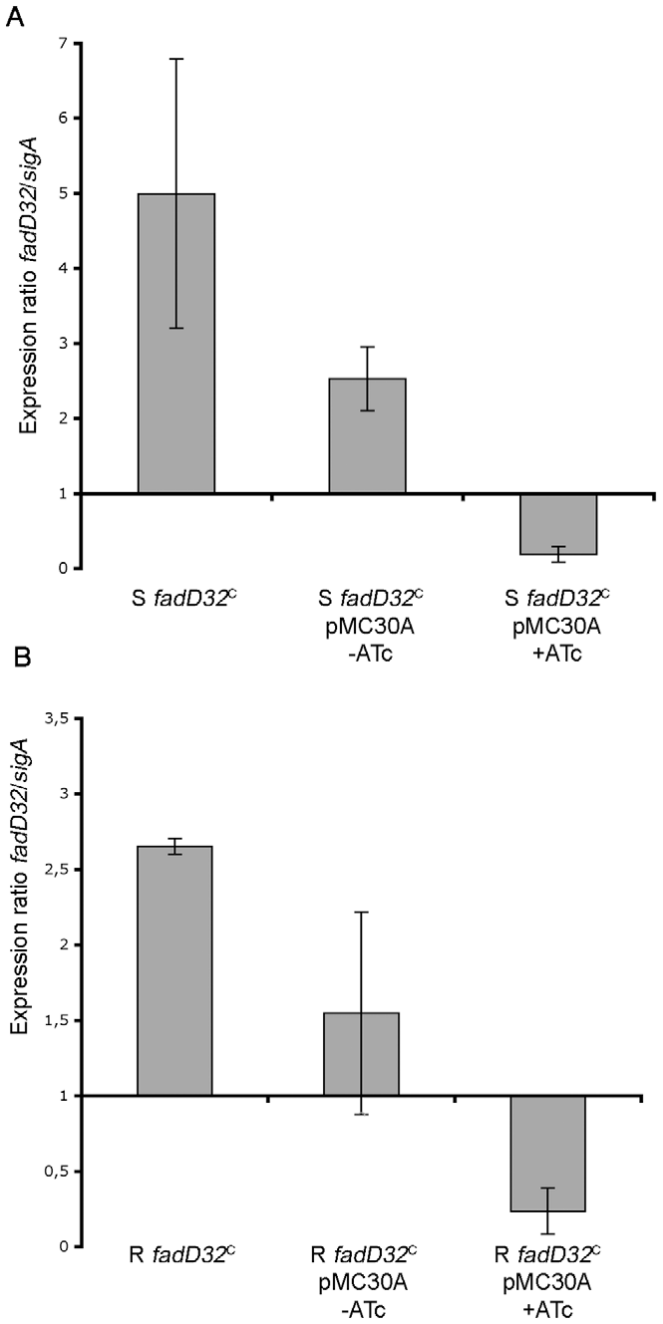
Quantification of *fadD32* expression in conditional mutant. Expression of *fadD32* was measured by quantitative real-time PCR as described in Materials and Methods, using *sigA* gene as a housekeeping gene standard. Results are relative expression ratios compared to expression in wild type strain (S or R). Expression was measured in wild type strain, *fadD32^C^* strains and *fadD32^C^* pMC30A strains with or without 200 ng/ml ATc after 48 h culture. (A) results in S *fadD32* ^C^ derivative strains; (B) results in R *fadD32* ^C^ derivative strains.

To confirm these results, the expression level of *fadD32* was measured by quantitative RT-PCR (qRT-PCR) in both *fadD32* ^C^ strains after 48 h with or without 200 ng/ml ATc. As shown in figure 6A, when *fadD32* was positioned under the P*_ptr_* promoter (strain S *fadD32* ^C^), its expression was 5-fold higher than with its own promoter. When the TetR/PipOFF system was introduced (via pMC30A) into this construct, the expression of *fadD32* was decreased but was still higher than in the wild type strain. In the presence of 200 ng/ml of ATc, qRT-PCR confirmed the total absence of fadD32 mRNA and by so the inhibitory activity linked to the TetR/PipOFF system. Similar results were obtained in the R strain (Figure 6B).

In conclusion, these experiments have demonstrated for the first time the functionality of the TetR/PipOFF regulated system in *M. abscessus* on *lacZ* and *fadD32* genes. We have confirmed that *fadD32* is also an essential gene in *M. abscessus*.

## Discussion

Ever since the recognition recently in 1992 of M. abscessus as a species, the number of clinical reports of infections due to M. abscessus has increased annually, mainly but not exclusively affecting subjects with predisposing conditions (e.g., Cystic Fibrosis, or those receiving immunosuppressive therapy). Aside from its role as an emerging pathogen, M. abscessus may function as a suitable surrogate host for the study of mycobacterial infections in general. Like M. smegmatis, it grows rapidly on conventional culture media and is a level 2 pathogen. However, it shares a number of traits with slow-growing mycobacterial pathogens, such as the formation of caseous lesions [13] and the ability to persist silently for decades in the human host [10]. Several relevant in vivo and ex vivo models of infections have already been developed [13], [14], [15], [33] and its complete genome sequence is now available [17].

However, a true understanding of the genetics of *M. abscessus* is still in its infancy with only one KO mutant described so far [18]. *M. abscessus* is indeed one of the most resistant of the mycobacteria, with a high frequency of spontaneous antibiotic-resistant clones when selecting for recombinants, which has hampered the use of common resistance markers. In addition, its metabolism and the optimal temperature for growth may differ between *M. abscessus* and other mycobacterial species. For example, the absence of a sucrose import system in *M. abscessus* would explain the lack of toxicity in the presence of sucrose; even if the *sacB* gene is expressed. This makes the thermosensitive counterselectable plasmid based on sucrose sensitivity inefficient [19]. In addition, *M. abscessus* does not grow above 39°C (the temperature for its optimal growth being 28°C), rendering the 40°C counterselection impossible. Finally, its S to R switch may modify the permeability of the outer layer, and the presence of a DNA degradation locus [34], a full plasmid and a complete phage [17] might restrict the efficiency of foreign DNA integration and/or recombination into the chromosome, either by DNA degradation or incompatibility.

Having taken into account all of these mycobacterial properties, we decided to test the recently developed TetR/PipOFF system, which has previously proven to be extremely useful in the detection and study of conditional mutants in *M. tuberculosis* and *M. smegmatis* [30], [35]. The TetR/PipOFF system allows repression to be achieved by the addition of ATc rather than its removal from the culture medium. The ATc based induction of the Pip-encoding gene permits a tight repression of the gene under investigation [30]. Several steps were necessary to adapt the TetR/PipOFF system to *M. abscessus*. Firstly, it was necessary to use different and integrative vectors coding for the zeocin resistance. This resistance marker was shown to be more efficient than the kanamycin resistance marker, by selecting far fewer spontaneous antibiotic-resistant mutants [18], and was used at a concentration of less than 50 µg/ml. The second step was to achieve a recombination event, by single cross-over. We firstly electroporated the recently developed mycobacterial recombinase-based system pJV53 [21] in *M. abscessus* S and R isotypes. Plasmid pMC18 was then electroporated in both S and R isotypes – pJV53 and integration by single cross-over was checked by amplification. The success of this gene replacement, which represents only the second published description so far in *M. abscessus*, has allowed us to demonstrate, once again, the efficacy of pJV53 and its recombinases in both isotypes. Finally, *lacZ* as a simple marker of expression, and *fadD32* as an example of an essential gene have confirmed the functionality of the adapted TetR/PipOFF in *M. abscessus*.

However if gene expression can be dramatically reduced in *M. smegmatis* and *M. tuberculosis* with 50 ng/ml and 200 ng/ml of ATc respectively ([30] and our study), we were unable to obtain a similar reduction in *M. abscessus*, even at the highest concentration of ATc (200 ng/ml). Previous studies have shown that the MIC for tetracycline is low in *M. smegmatis*, but higher in *M. tuberculosis*, thus explaining the use of a higher ATc concentration to induce the TetR repression in *M. tuberculosis* [25]. Less is known about resistance of *M. abscessus* towards tetracycline. Several studies have previously shown that *M. abscessus* possesses a number of resistance determinants, which makes this bacterium more resistant than *M. smegmatis* or *M. tuberculosis* towards ATc [36]. We have also identified a difference between S and R strains, the R isotype showing a higher level of *lacZ* repression for similar ATc concentrations, compared to S strain. A number of recent studies have shown that the S/R switch in *M. abscessus* was associated with the loss of a surface molecule named glycopeptidolipid (GPL) [13], [15], [33], [37]. The difference between the S and R strains of *M. abscessus* might thus be due to changes of surface properties and the degree of permeability from the loss of GPL. However, nothing is known regarding comparative MICs towards ATc for both isotypes. In addition, the induction of efflux systems was demonstrated to be responsible for the lower efficiency of ATc used at high concentrations [25].

Several leads might enable us to improve the adapted TetR/PipOFF system, for example by isolating highly efficient *M. abscessus* strains for electroporation, as described several years ago for *M. smegmatis* with the isolation of the mc^2^155 strain [38]. R strains form aggregates and tend to stick to plastic surfaces, which hampered electroporation efficiency. The other improvement might be the counter selective marker. One recently developed antibiotic, Nourtheothricin (http://www.webioage.com/) (Jun-Rong Wei and Eric Rubin, personal communication) was shown to be active against *M. abscessus*, and might represent only the second efficient counterselective agent to be developed for use as a genetic tool for *M*. *abscessus*. This further demonstrates the difficulties encountered when working on *M. abscessus*, and henceforth might impede the development of genetic tools dedicated to this species.

Using the TetR/PipOFF system, we have demonstrated the essential role of the *fadD32* in *M. abscessus*. Although predictable - *fadD32* does plays an important role in the formation of the outer membrane and in cell-wall permeability [39] making it essential for *M. tuberculosis* and *M. smegmatis* [32] *-* this result nonetheless proves the functionality of the adapted TetR/PipOFF system in *M. abscessus*. This will permit further investigation of candidate genes, such as those identified recently using a transcriptomic approach to unravel the molecular mechanisms of the S/R switch (Herrmann et al., unpublished, and [16]). Indeed, while we know that the S/R switch is essential for *M. abscessus* to modulate the innate immune response of the host [13], [16], nothing is currently known about the precise mechanisms involved. The development of the TetR/PipOFF system may also allow the characterization of the respective roles of candidate virulence genes recently identified in the *M. abscessus* genome [17].

## Materials and Methods

### Bacterial strains, transformation condition and growth conditions

Bacterial strains and plasmids are described in table 1 and on figure 1. Bacteria were grown in Luria-Bertani (LB) broth at 37°C with agitation. Ampicillin (100 µg/ml, Sigma, Saint-Louis, MO, USA), kanamycin (50 µg/ml, Sigma, Saint-Louis, MO, USA), zeocin (25 µg/ml for *E. coli*; 50 µg/ml for mycobacteria, Invitrogen, Paisley, UK), hygromycin (200 µg/ml, Roche Diagnostics, Mannheim, Germany), streptomycin (50 µg/ml, Sigma, Saint-Louis, MO, USA) and X-gal (50 µg/ml, MP biomedicals, Illkirch, France) were added when necessary. ATc (Sigma, Saint-Louis, MO, USA) was added as required at concentrations starting at 0 and up to 300 ng/ml. Preparation of electrocompetent cells, electroporation and preparation of mycobacterial genomic DNA were performed as previously described [40], [41]. The plasmid pJV53, which express recombinases [21], was electroporated in *M. abscessus*. This plasmid is used to induce the crossing-over as described previously [10]. After several sub-cultures in the absence of a selective marker, the pJV53 plasmid can be lost by the strain, as checked by kanamycin sensitivity.

### Plasmids construction

Restriction endonucleases and modification enzymes (phosphatase and Klenow fragment) (New England Biolabs, Ipswich, UK) were used according to the manufacturer's instructions. PCRs were performed using 1 U DyNazyme DNA polymerase (Finnzymes, Espoo, Finland) in 1X buffer, 200 µM concentration of each deoxynucleoside triphosphate (MP biomedicals, Illkirch, France), 0.8 µM concentration of each primer (Eurogentec, Seraing, Belgium), and 10 ng of chromosomal DNA in a 50-µl reaction volume. Cycling conditions were as follows: 1 cycle of 5 min at 94°C; 30 cycles of 20 s at 94°C, 20 s at 55°C, and 40 s/kb at 72°C; with a final extension of 10 min at 72°C. PCR products were separated in 1% agarose gels for 1 h at 10 V/cm of gel. DNA fragments were purified from agarose gel by use of a Wizard® SV gel and PCR Clean-Up system (Promega, Madison, WI, USA). *E. coli* DH5α strain (Invitrogen, Paisley, UK) was used for cloning experiments.

Plasmid pMV*lac* was constructed starting with the restriction of pMV306 plasmid by *Eco*RV followed by the ligation with the PCR product P*_ptr_*-*lacZ* purified after amplification using primers MC77 and *lac*Z-3 using plasmid pFRA42A as target (Figure 1 and S2 and Table 1 and 2). Plasmid pMC30A (Figure 1 and S2) was constructed as a derivative of pMV*lac* after restriction by *Nco*I followed by ligation of the restricted fragments P*_furA102_tetO-pip* and P*_smyc_-tetR* both purified after *Sph*I-*Eco*RI digestion of pFRA61 plasmid (Table 1). Plasmid pMC30B (Figure 1 and S2) represents the opposite of pMC30A with P*_furA102_tetO-pip* and P*_smyc_-tetR* ligated in opposite orientation in the *Nco*I site of pMV306 plasmid (Table 1).

Zeocin, as a selective marker, was inserted by amplification with primers ZeoF and ZeoR from the plasmid pLYG204-Zeo, then restricted by *Spe*I and ligated into the plasmid pFRA50 previously restricted by *Spe*I and purified, to create pMC14 (Table 1). 953 bp of the 5′ end of *fadD32* (MAB_0179) was amplified using primers fadF and fadR (Table 2) and then cloned into the *Nsi*I site of pMC14 which is located downstream of the P*_ptr_* promoter giving plasmid pMC18 (Table 1 and Figure S1).

### Construction of conditional mutants in *M. abscessus*

Plasmid pMC18 (Table 1), which contains the first 953 bases of *fadD32* (MAB_0179) under the control of the P*_ptr_* promoter, was electroporated into *M. abscessus* strain bearing pJV53 (Table 1). After a single cross-over event, pMC18 was integrated into the chromosome giving the entire *fad32* gene under the control of P*_ptr_* promoter and a deleted part of *fad32* gene under the control its own promoter (strain *fadD32* ^C^, see Table 1). *M. abscessus* recombinants were isolated afterwards on LB agar plates after zeocin selection (50 µg/ml). Plasmid pMC18 integration by single cross-over was checked by amplification using primers MC77 (on P*_ptr_* promoter) and fadV (on 3′end of *fadD32*) (Table 2). After elimination of plasmid pJV53, plasmids pMC30A and pMC30B (Table 1) were electroporated to obtain the final conditional mutant *fad32*. After kanamycin (50 µg/ml) and X-gal (50 µg/ml) selection on LB agar, strains S or R *fadD32* ^C^-pMC30A or –pMC30B were obtained. Transformation with pMV*lac* (Table 1) was performed (strains S or R *fadD32* ^C^-pMVlac) to control for functionality of the plasmid without the two regulatory genes *pip* and *tetR*.

### β-galactosidase activity assay

β-galactosidase activity assay was performed as previously described and enzymatic activity expressed in Miller units [42]. For *M. abscessus* R strains, OD_600 nm_ was taken after sonication to represent a homogenous suspension. Exponentially growing culture cultivated at 37°C with agitation were diluted in new media in the presence of 0, 100, 200, or 300 ng/ml of ATc and incubated in similar conditions. At 24, 48 and 72 h, an aliquot was taken to perform β-galactosidase activity assay. Each assay was performed in triplicate. Results were expressed as the relative percentage of Miller unit for the tested strain at a give time versus Miller unit for the tested strain in the absence of ATc.

Assays on *M. smegmatis* mc^2^155 derivative strains were performed on culture prepared like described by Boldrin et al. [30]. Briefly, cultures in liquid medium were grown 18 h with or without ATc (50 ng/ml). After, culture without ATc was diluted in new culture +/− ATc (50 ng/ml), and culture with ATc was diluted in same condition (with ATc). After 24 h, enzymatic activity of β-galactosidase was measured. Each assay was performed in triplicate.

### Growth analysis

10-fold serial dilutions were made from exponentially grown cultures (37°C with agitation) in the absence of ATc, and 5 µl of each dilution was plated on LB agar in the presence or absence of ATc (200 ng/ml). After five days, the presence of growth was checked for each condition and comparison was performed between plate with or without ATc. Each assay was performed in triplicate.

### RNA extraction

Total RNA was extracted from 10 ml of bacteria in liquid culture (with or without ATc at 200 ng/ml). Pellet of bacteria was mixed with 1 ml of Trizol (Invitrogen, Paisley, UK) and placed in lysing matrix B tubes (MP Biomedicals, Illkirch, France). Bacteria were lysed by shaking in a Fastprep FP120 apparatus (MP Biomedicals, Illkirch, France). Two hundred microliters of 1-bromo-3-chloro-propane were added and tubes were centrifuged for 10 min at 10 000 g. The aqueous phase was recovered, and RNAs were precipitated with 500 µl of isopropyl alcohol and washed with ethanol 75%. RNAs were then resuspended in 50 µl of water, treated with DNase I, and purified on RNeasy minicolumns (Qiagen). The quantity and the quality of the RNAs were verified spectrophotometrically (Nanodrop ND-1000) and on agarose gels.

### qRT-PCR

Reverse transcriptase PCR (RT-PCR) was performed with Superscript RT II (Invitrogen) using gene specific primer (MC83 and MC85, Table 2). The primer sequences were designed with Primer3Plus online software [43]. Samples without RT were prepared concurrently and analysed for the absence of contaminating genomic DNA. Real-time quantitative PCR analysis was performed using FastStart SYBR Green Master (Roche Diagnostics, Mannheim, Germany). cDNAs obtained as described above were diluted ten-fold in nuclease-free water before qPCR. The PCR program consisted of 35 amplifications/quantification cycles of 95°C for 15s and 60°C for 1 min, with signal acquisition at the end of each cycle. MC82 and MC83 were used to amplify *sigA* and MC84 and MC85 to amplify *fadD32* (Table 2). Equation 1 from Pfaffl [44] was used to analyse the statistical significance of the data, using *sigA* as a reference gene standard. Each assay was performed in duplicate and repeated at least twice.

## Supporting Information

Figure S1 TetR/Pip OFF system mechanism and function of plasmids used in this system.
(TIF)

Figure S2 Construction of pMC18, pMV*lac*, pMC30A and pMC30B.
(TIF)
