# Supplementary figures and images for: Conditional Gene Expression in Mycobacterium abscessus
Source: PLoS One. 2011 Dec 15;6(12):e29306. doi: 10.1371/journal.pone.0029306 (PMC3240655; doi:10.1371/journal.pone.0029306)

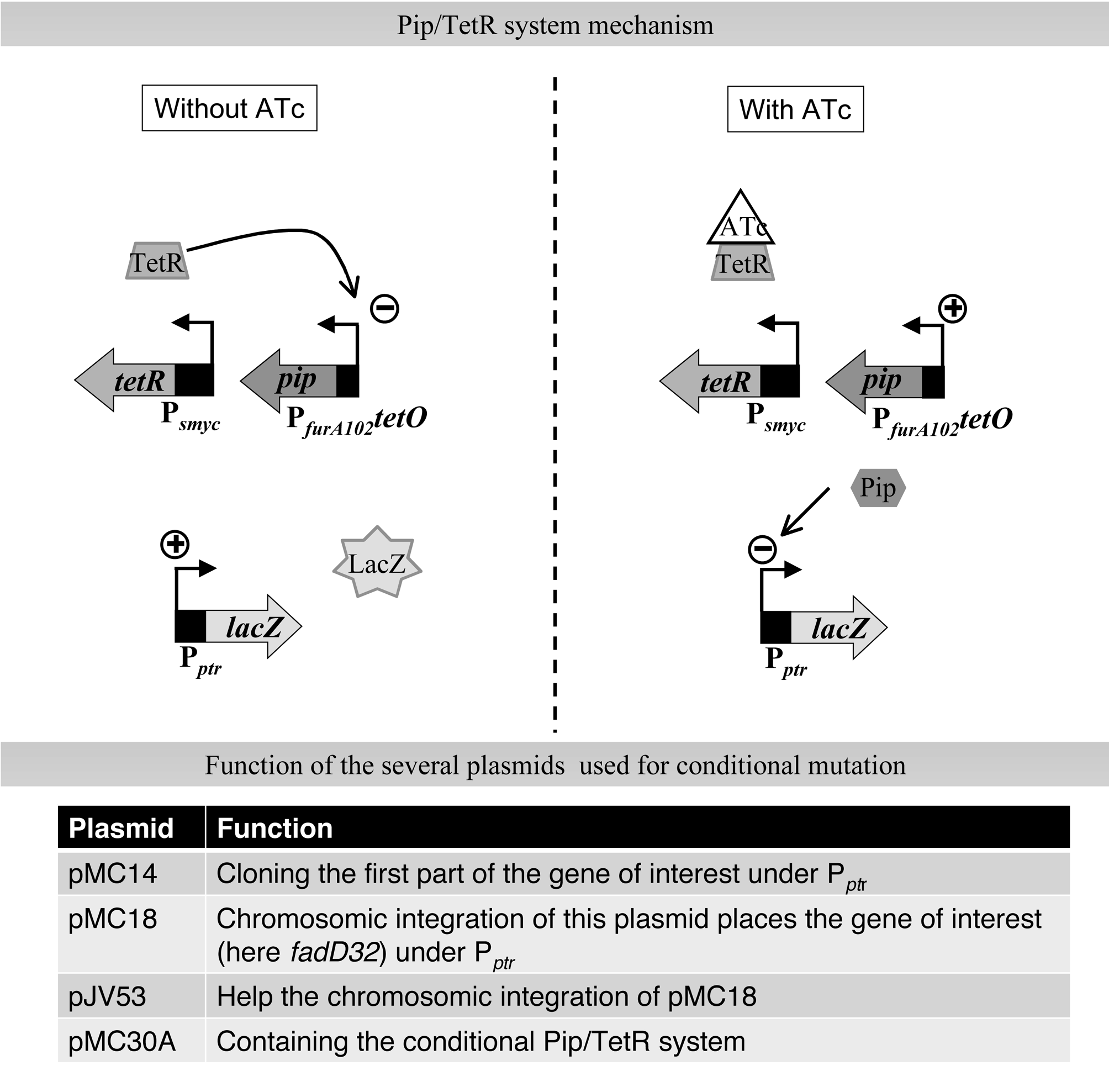

Supplement: Figure S1 — TetR/Pip OFF system mechanism and function of plasmids used in this system. (TIF) [file pone.0029306.s001.tif]

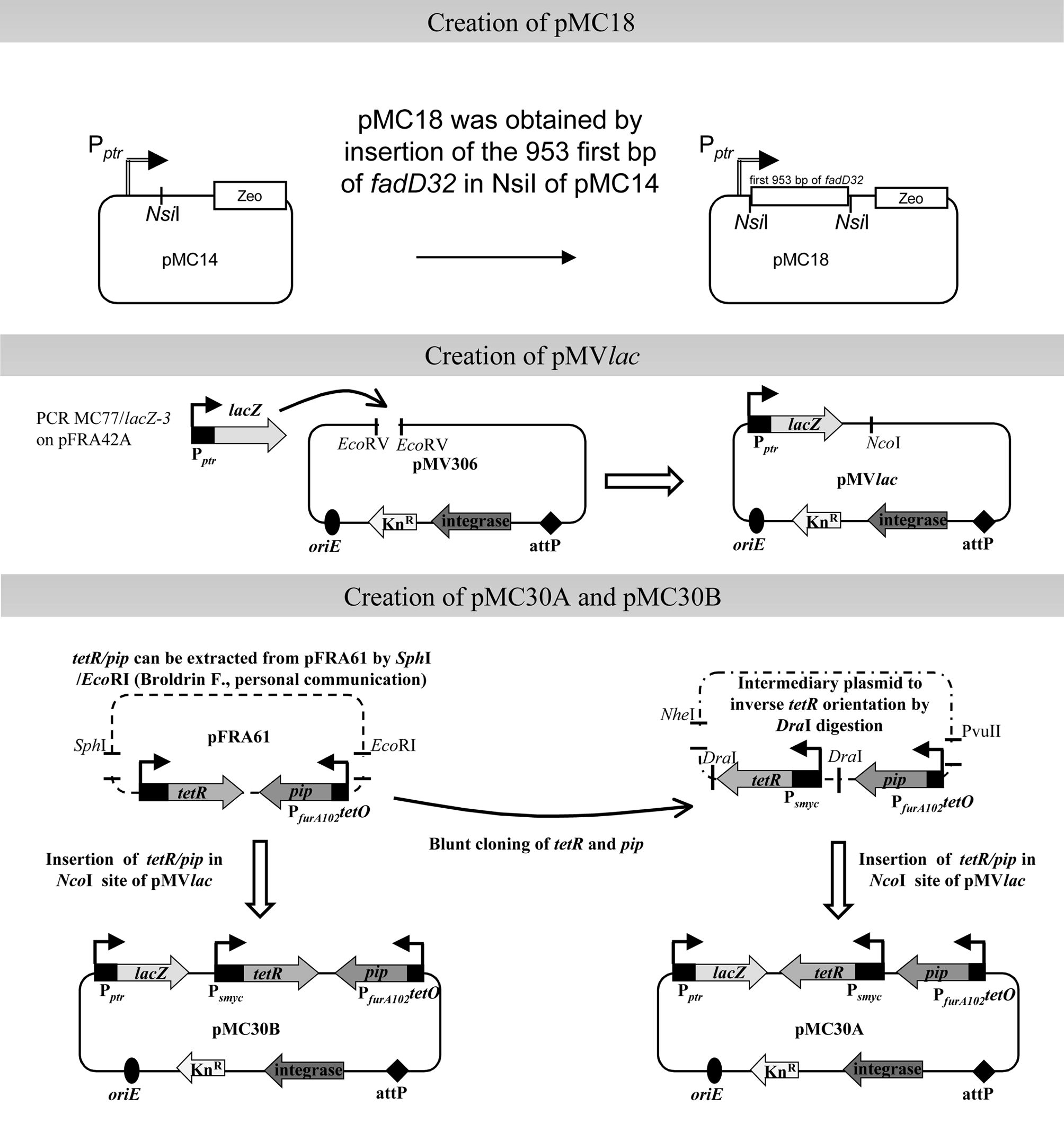

Supplement: Figure S2 — Construction of pMC18, pMVlac, pMC30A and pMC30B. (TIF) [file pone.0029306.s002.tif]
